# Lived Experiences, Disease Management and Expectations in a Nursing-Led Psoriasis Unit: A Qualitative Study

**DOI:** 10.3390/healthcare14121647

**Published:** 2026-06-10

**Authors:** Elena Violeta Iborra-Palau, Elena García-Redondo, Carlos Blasco-García, Raquel Alabau-Dasi

**Affiliations:** 1 Deparment of Nursing and Podiatry, University of Valencia, 46010 Valencia, Spain; elena.iborra@uv.es (E.V.I.-P.); carlos.blasco@uv.es (C.B.-G.); 2 Malvarrosa Hospital, 46011 Valencia, Spain; gare@alumni.uv.es

**Keywords:** psoriasis, phototherapy, life change events, cost of illness, patient compliante, nurse-patient relations, treatment burden

## Abstract

**Highlights:**

**What are the main findings?**

- Phototherapy in psoriasis operates as a “double-edged sword”, where its clinical effectiveness is counterbalanced by a substantial logistical and psychosocial burden that may exacerbate disease activity.
- Patients experience a profound biographical disruption, driven by visible lesions, chronic symptoms, and an “adherence–stress cycle”, alongside significant gaps in knowledge about systemic comorbidities.

**What are the implications of the main findings?**

- These findings highlight the need to move beyond skin clearance toward integrated, patient-centred care models that address psychosocial, educational, and logistical dimensions of the disease.
- Establishing a specialised nursing referent within dermatology units is essential to improve patient navigation, treatment adherence, and overall quality of life.

**Abstract:**

**Background:** Psoriasis is a chronic systemic disease affecting over 60 million people. While phototherapy is effective, its demanding schedule imposes a significant treatment burden. This study explores the lived experiences and healthcare expectations of patients in specialized nursing-led phototherapy units. **Methods:** A descriptive phenomenological study was conducted (2019–2022) with 72 participants at a Spanish tertiary hospital. Data from semi-structured interviews were analyzed using inductive thematic content analysis. **Results:** Nine subtopics emerged within four main thematic areas: (1) a gap between psychological awareness and low knowledge of systemic comorbidities; (2) psychological stress as the primary disease trigger; (3) an adherence-stress cycle causing biographical disruption; and (4) a collective demand for a permanent nursing referent to overcome systemic barriers. **Conclusions:** Phototherapy’s clinical efficacy is often undermined by its logistical rigor. Achieving skin clearance is insufficient if biographical and systemic gaps remain. Integrated care models, led by specialized dermatology nurses, are essential to provide clinical navigation and support beyond conventional skin treatment.

## 1. Introduction

Psoriasis is a chronic, immune-mediated, and inflammatory disease. According to the latest Global Psoriasis Atlas, it affects over 60 million people worldwide, with prevalence rates reaching 11.4% in high-income countries [1].

Traditionally, psoriasis has been viewed as a dermatological condition; however, recent evidence supports its classification as a systemic disease with associations beyond the skin [2]. Psoriasis is frequently associated with comorbidities such as cardiovascular disease, metabolic syndrome, non-alcoholic fatty liver disease, obesity, and other immune-mediated diseases [3,4]. Beyond physical manifestations, patients with psoriasis report symptoms of anxiety and depression, often exceeding the psychological distress seen in other chronic conditions [5].

The clinical presentations of psoriasis are highly heterogeneous. Lesions can affect any part of the body, including the scalp, nails, and genital areas [6]. These skin lesions, which are usually visible, have a significant impact on self-concept and relationships with others [7]. Shame, low self-esteem, and social withdrawal are common, often affecting daily functioning and quality of life [8,9]. This experience is frequently interpreted through the lens of biographical disruption [10]. In this framework, the chronic condition forces individuals to redefine their identities and social roles. This disruption is further exacerbated by elevated rates of suicidal ideation and psychiatric distress, as highlighted in a recent systematic review [11].

Among the available treatments for moderate-to-severe psoriasis, phototherapy is an effective therapeutic option [12,13]. However, from a patient-centered perspective, this treatment imposes a significant treatment burden. The requirement for thrice-weekly in-hospital sessions demands substantial time and effort. Furthermore, clinical effectiveness is strictly contingent upon high adherence rates [14]. The rigid logistics of hospital-based therapy continue to create significant friction in work–life balance. This stress often triggers emotional distress, which may paradoxically exacerbate clinical flares [15]. Despite its known benefits, the treatment burden experienced by patients has not been explored in depth, and understanding how to approach this type of treatment requires special attention.

Nursing professionals in dermatology units are essential for bridging the gap between clinical severity and patient well-being. Beyond the technical administration of ultraviolet radiation, nurses provide complex interventions focused on health literacy, patient education, and psychosocial support. These nurse-led interventions are now recognized as fundamental components of comprehensive psoriasis care [16]. Despite the importance of the nursing role in patient education, many patients still harbor misconceptions about the disease, including fears of contagion, chronicity, or treatment options [17]. Zhang et al. emphasize that many patients still possess an insufficient understanding of disease triggers, genetic factors, and the potential side effects of systemic treatments [18]. This gap is critical, as increased knowledge has been shown to correlate positively with more favorable attitudes and effective self-management practices [19].

Quantitative research has thoroughly analyzed adherence rates and clinical outcomes [20]. This includes large-scale, influential studies such as the LITE Randomized Clinical Trial [21]. Despite these advances, a significant knowledge gap remains regarding the qualitative, lived experience of treatment burden. The recent literature continues to prioritize clinical metrics or statistical surveys on patient attitudes [11,18], leaving the subjective experience within the organizational context of nurse-led units underexplored. Understanding how patients cope with the emotional and logistical complexities associated with treatment is not a mere contextual detail; it is fundamental to defining the scope of dermatological nursing. Exploring these experiences is essential to transforming phototherapy units from technical treatment spaces into integrated, patient-centered models. Therefore, this study aims to explore the lived experiences and disease management strategies of people with psoriasis undergoing phototherapy to strengthen the rationale for specialized nursing interventions.

## 2. Materials and Methods

### 2.1. Design

A qualitative study was conducted using a descriptive phenomenological approach. This design allowed for an in-depth exploration of the first-person experiences of the phenomenon under study, namely, the treatment burden and disease management strategies of people with psoriasis undergoing phototherapy. The study was situated within Husserl’s descriptive paradigm, recognizing that the health–illness process is a subjective reality constructed through the personal and social contexts of the participants [22].

While the epistemological foundation of the study was phenomenology, inductive thematic analysis was employed as a systematic method for organizing and synthesizing the findings from a large dataset. This hybrid approach ensured that the lived experience remained central to the inquiry while providing a structured framework for data analysis.

Although this study represents the qualitative component of a larger mixed-methods research project, it is presented here as a robust, independent analysis of patients’ subjective narratives, providing a comprehensive understanding of the psychosocial impact of phototherapy.

### 2.2. Setting and Participants

The study was conducted in a nurse-led phototherapy unit within a large tertiary-level public university hospital in Spain. As a regional referral center for complex dermatological conditions, the facility manages a high volume of specialized care and substantial healthcare demand. Its broad geographical catchment area, encompassing both metropolitan and rural populations, results in a high patient turnover that facilitated the recruitment of a large and diverse sample.

A purposive and exhaustive sampling strategy was employed. Participants were recruited from the larger parent research project. The approach was purposive because it targeted patients with moderate-to-severe psoriasis and exhaustive because every eligible individual from the parent cohort between 2019 and 2022 was invited to participate.

Four inclusion criteria were applied: (1) age ≥ 18 years; (2) a confirmed diagnosis of psoriasis; (3) current receipt of phototherapy treatment; and (4) the ability to provide informed consent and participate in an in-depth interview. Patients with other dermatological conditions or cognitive impairments were excluded. Cognitive impairment was defined as pre-existing neurological or psychiatric conditions (e.g., Alzheimer’s disease or severe cognitive decline) that could impede the participant’s ability to provide valid informed consent or engage in a sustained and coherent semi-structured interview. This was assessed through a review of clinical records and a preliminary evaluation of communicative competence conducted by the primary researcher during the recruitment phase.

The final sample consisted of 72 participants recruited between 2019 and 2022. No eligible participants declined to participate. Although the sample size exceeded the range typically reported in descriptive phenomenological studies, it was justified by the objective of achieving high information power. This concept, proposed by Malterud et al., has been suggested as an alternative to the traditional concept of data saturation introduced by Glaser and Strauss when determining sample size in qualitative research [23,24].

In the present study, the sample was considered adequate for the phenomenon under investigation because participants represented a wide range of heterogeneous life circumstances. The inclusion of a substantial number of participants allowed for the exploration of diverse existential contexts, ranging from individuals in early adulthood with a recent diagnosis of psoriasis to those living with the disease for many years, as well as participants with differing family structures, caregiving responsibilities, and occupational demands.

This diversity contributed to high information power by enabling different participant profiles to enrich the findings in distinct ways. For example, individuals with long-standing psoriasis provided deeper insights into the relationship between treatment adherence, stress, and biographical disruption, whereas newly diagnosed participants contributed more substantially to the theme of limited disease awareness. To ensure sufficient analytical depth across such a large sample, data analysis followed an iterative process involving detailed individual case analysis followed by cross-case synthesis.

This broad and heterogeneous sample ensured not only the achievement of data saturation but also its verification across different participant profiles, allowing the identification of robust and consistent patterns of experience across diverse life-worlds.

### 2.3. Data Collection

Data were collected between December 2019 and November 2022 through individual, semi-structured face-to-face interviews. The interviews were conducted by the nurse (PhD) responsible for the phototherapy service. Although the participants were already familiar with her in a clinical capacity, they were unaware of her personal goals and motivations regarding the research. She also served as the principal investigator of the project.

To mitigate potential social desirability bias, participants were explicitly informed that the interviews were conducted solely for research purposes, were independent of their clinical care, and that their responses would not affect their treatment in any way.

The semi-structured interview guide was developed by the research team following a comprehensive review of the literature on psoriasis treatment burden. To ensure face validity and clinical relevance, the guide was reviewed by two senior dermatology researchers and subsequently piloted with the first three participants to refine the wording, clarity, and flow of the questions.

The final interviews were structured around four predefined guiding themes: (1) understanding of psoriasis and treatment options; (2) perceived triggers of flare-ups; (3) disease-related stressors; and (4) suggestions for improving healthcare services.

Interviews lasted between 30 and 40 min and were conducted in a private room within the hospital, separate from the treatment area. All interviews were audio-recorded with participants’ verbal and written consent for subsequent analysis.

Throughout the data collection process, the interviewer maintained a field journal in which she documented reflexive observations and relevant aspects of participants’ non-verbal communication. In addition, sociodemographic data were collected, including age, sex, marital status, employment status, history of mental illness, and difficulties attending phototherapy sessions.

These variables were collected to contextualize participants’ life-worlds and to facilitate the exploration of how different social and occupational roles influenced the perceived treatment burden and experiences of biographical disruption.

### 2.4. Data Analysis

All audio-recorded interviews were transcribed verbatim by the primary investigator to ensure deep immersion in the data, avoiding the use of automated transcription software. The transcripts were subsequently imported into NVivo qualitative data analysis software (Version 12 Pro, QSR International Pty Ltd.) to facilitate a systematic and transparent coding process [25].

The operationalization of information power followed Malterud’s five criteria: study aim, sample specificity, use of established theory, quality of dialogue, and analysis strategy [24]. Through an iterative analytical process conducted concurrently with data collection, data richness was continuously assessed until the core thematic categories demonstrated structural stability across the diverse participant profiles.

Data analysis followed a multi-stage inductive thematic approach, consistent with the principles of naturalistic inquiry, which served as the procedural framework for organizing and synthesizing the phenomenological findings. First, repeated readings of the transcripts enabled immersion in the data and the identification of meaningful units. Second, a cross-case analysis was conducted to synthesize these units into broader themes, ensuring that categories emerged inductively from participants’ narratives rather than from preconceived theoretical frameworks [26].

NVivo was used not only as a coding tool but also as a data management platform to enhance auditability and traceability throughout the analytical process. By applying classification attributes (e.g., disease duration and occupational burden), the research team systematically examined the consistency of themes across different participant profiles. Analytical memos were maintained throughout the analysis to document emerging interpretations and phenomenological insights.

The interviewer’s specialized clinical background further contributed to the establishment of high-quality dialogue, thereby maximizing the study’s information power. Finally, the initial analysis conducted by the primary researcher underwent investigator triangulation. An experienced dermatology nurse independently reviewed the coding framework, and any discrepancies were resolved through discussion until consensus was reached.

### 2.5. Rigor and Trustworthiness

Several strategies were employed to ensure the credibility, confirmability, transferability, and dependability of the findings [27].

Credibility was enhanced through two key strategies. First, the findings were reviewed through a peer debriefing process involving a second, senior dermatology nurse, who examined the coding framework and transcripts to ensure that the emergent themes were firmly grounded in the data. Second, a form of member checking was conducted whereby participants received feedback on the interview findings to verify that the interpretations accurately reflected their intended meanings.

To ensure confirmability and actively manage potential researcher bias, a rigorous reflexive process was maintained throughout the study. We acknowledge that the principal investigator’s dual role as both interviewer and analyst may have introduced bias. To minimize this risk, the researcher engaged in bracketing (epoché) through the use of a reflexive journal, documenting and setting aside potential clinical assumptions and preconceptions before and after each interview.

This process of internal reflexivity, combined with the external scrutiny provided through peer debriefing by senior researchers who had no prior clinical relationship with the participants, helped ensure that the findings remained grounded in and faithful to participants’ narratives.

Transferability was supported through detailed descriptions of the study context and participant characteristics, enabling readers to assess the applicability of the findings to other settings. Dependability was enhanced through the use of a consistent and transparent research process, including verbatim transcription of interviews, duplicate audio recordings, and the maintenance of field notes to capture contextual information and relevant observational details.

### 2.6. Ethical Considerations

The study received approval from the Ethics Committee of the University General Hospital of Valencia (Approval No. 7/2019). Ethical approval was maintained throughout the study period through the submission of mandatory annual progress reports to the ethics committee, in accordance with institutional requirements.

All participants provided informed consent prior to participation. Identifiable data were anonymized, and audio recordings were permanently deleted following transcription.

Given the sensitive nature of discussions surrounding anxiety, stress, and the challenges of balancing treatment, disease management, and social, family, and occupational responsibilities, particular attention was paid to participants’ emotional well-being during the interviews. The researcher was prepared to pause or postpone any interview if a participant became emotionally distressed.

However, participants who experienced emotional distress during the interviews expressed a preference to continue, often reporting that the opportunity to discuss their experiences was itself therapeutic. To further safeguard participants’ well-being, dermatologists from the unit were available to facilitate referral to mental health services when deemed necessary.

## 3. Results

To present the findings, all participant responses were anonymized to ensure confidentiality. Direct quotations are included to illustrate key findings and are identified using a participant code (e.g., P41, female, 22), indicating anonymous participant identification, gender, and age. In addition, the demographic characteristics of the participants are presented in Table 1 to provide contextual information about the sample.

In keeping with the principles of qualitative research, numerical quantifiers were intentionally avoided in the reporting of thematic findings to ensure that the focus remained on the richness and depth of participants’ experiences rather than on the frequency with which particular views were expressed.

**Figure 1:**
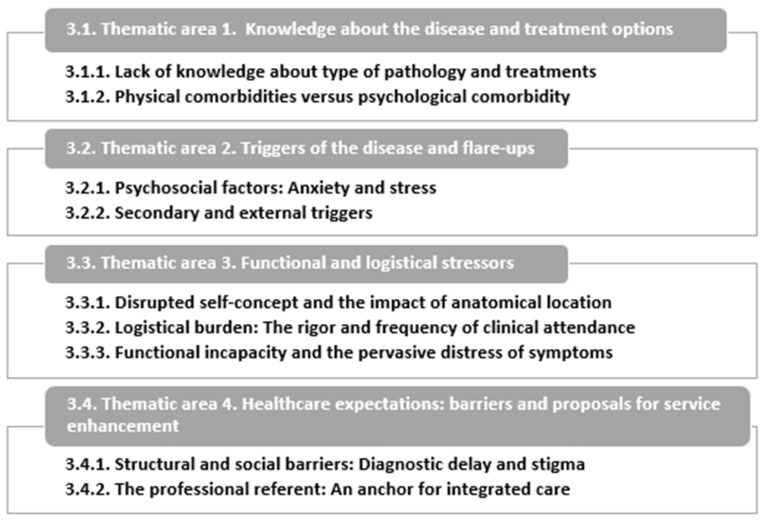
Thematic Areas and Subtopics.

Inductive analysis of the interviews revealed a complex interaction between psoriasis treatment and patients’ lived experiences. The findings were organized into four overarching thematic areas that corresponded to the central topics explored in the interview guide. Within these thematic areas, nine subthemes emerged inductively from the data. Figure 1 provides a summary of the thematic areas and their corresponding subthemes.

The results are presented below, structured according to the four main thematic areas.

### 3.1. Thematic Area 1: Knowledge of the Disease and Treatment Options

This thematic area explores participants’ understanding of psoriasis and available treatment options, highlighting a disparity between their recognition of the psychological burden of the disease and their limited awareness of its systemic comorbidities.

#### 3.1.1. Lack of Knowledge About the Disease and Available Treatments

Participants’ knowledge was generally insufficient to support effective self-management and empowerment. Regarding the chronic nature of psoriasis, several participants were unaware or uncertain that the disease is incurable:

“Well, I know that what I have is chronic, right?”

(P41, female, 22).

This lack of awareness extended to concerns about contagion, leading to feelings of shame, social withdrawal, and avoidance of close contact with others:

“I was really ashamed… My brother had a daughter, and I didn’t even pick her up because I thought, ‘What if she catches it?’… I didn’t even know what it was. I didn’t even know it was psoriasis until I came here in August. I always thought it was eczema.”

(P55, female, 41).

Significant knowledge gaps were also evident regarding treatment alternatives when first-line therapies proved ineffective:

“He [the doctor] prescribed acitretin, which was the best option at the time, but then it stopped working, and I don’t know what other treatment options are available for psoriasis.”

(P4, female, 71).

Furthermore, even participants who demonstrated greater knowledge about the disease expressed considerable concerns regarding biological and immunosuppressive therapies, often associating them with an increased risk of cancer:

“I know that there are injections. The doctor wanted to prescribe them to me, but I refused because they work by lowering the immune system and make you more likely to develop cancer.”

(P45, male, 51).

#### 3.1.2. Physical Comorbidities Versus Psychological Comorbidity

A striking disparity emerged between participants’ awareness of the psychological and physical consequences of psoriasis. While participants consistently acknowledged the close relationship between psychological well-being and disease activity, awareness of systemic physical comorbidities remained limited.

Many participants reported becoming aware of conditions such as cardiovascular risk, weight gain, or fatty liver disease only through educational interventions provided during phototherapy treatment:

“You were the one who told me that psoriasis tends to make people gain weight. I had no idea; you were the one who told me. And I didn’t know about fatty liver disease either—no one had ever told me about it.”

(P11, female, 62).

In contrast, participants demonstrated a profound understanding of the psychological burden associated with psoriasis. They described a bidirectional relationship between emotional distress and symptom exacerbation, forming what many perceived as a self-perpetuating cycle:

“It’s like a snake biting its own tail… If I have anxiety, it makes me itch; then I scratch, and that gives me even more anxiety.”

(P55, female, 41).

Even participants who were unaware of potential physical comorbidities reported experiencing a substantial psychological impact:

“No, I have no information about whether psoriasis can affect me physically through other diseases, but psychologically it definitely affects me.”

(P66, male, 34).

### 3.2. Thematic Area 2: Triggers of Disease Activity and Flare-Ups

Beyond their limited awareness of the systemic nature of psoriasis, participants identified a wide range of factors that triggered disease flare-ups. Their narratives revealed a clear hierarchy of perceived triggers, in which psychosocial factors were considered more influential than external or physical factors.

#### 3.2.1. Psychosocial Factors: Anxiety and Stress

Stressful life events were consistently perceived as the primary drivers of disease onset and exacerbation. Participants linked flare-ups to major life stressors, such as bereavement and serious illness, as well as to ongoing emotional strain:

“I associate the onset of the disease with stress. I am absolutely certain of this because there was a particularly critical moment in my life when my father died from advanced Alzheimer’s disease. He died young, and at the same time my mother was diagnosed with Hodgkin’s lymphoma.”

(P82, female, 57).

Beyond acute traumatic events, participants also described cumulative stress arising from work demands, family responsibilities, and everyday pressures as important triggers of symptom worsening:

“I think it happens when I’m very stressed, when I’m very anxious, or when I’m too busy and never stop to take a break.”

(P53, female, 41).

#### 3.2.2. Secondary and External Triggers

Although reported less frequently, external factors were also perceived as contributors to disease flare-ups. However, participants generally regarded these factors as secondary to psychological stress in the long-term management of psoriasis. Reported triggers included infections and physical trauma, consistent with the Koebner phenomenon.

“Well, I notice that the skin lesions appear when I get a sore throat or a severe throat infection.”

(P27, female, 37).

“What I do remember is that while playing football, I suffered a large wound, and when it was almost healed, psoriasis appeared on the scab. After that, it started to appear in other areas as well.”

(P62, male, 43).

### 3.3. Thematic Area 3: Functional and Logistical Stressors

The identification of these triggers contributed to a broader understanding of the functional and logistical distress embedded within the lived experience of psoriasis. This thematic area explores how chronic symptoms and treatment-related commitments generate significant biographical disruption, affecting participants’ personal, social, family, and professional lives.

#### 3.3.1. Disrupted Self-Concept and the Impact of Anatomical Location

Psoriasis profoundly affected participants’ self-perception, often leading them to avoid social interactions or physical activities because of fear of judgment and stigmatization:

“Psychologically, it’s the way it looks, seeing myself like this, seeing my legs the way they are. I really enjoy exercising, but when you wear shorts, people immediately look at your legs, and it affects me. For example, the other day at the gym, a girl working out next to me kept looking at them [my legs].”

(P45, male, 45).

The emotional impact was particularly pronounced among participants whose occupations involved direct interaction with others. Visible lesions on the hands were described as a source of embarrassment and emotional distress:

“I work with blind children. We use our hands to communicate all the time, and when I take them by the hand, they say to me, ‘Oh, you have wounds on your hands.’ My hands feel rough to them, and emotionally, it affects me.”

(P70, female, 57).

The anatomical location of lesions played a crucial role in shaping participants’ experiences. Lesions affecting highly visible or sensitive body areas were perceived as more stigmatizing than conditions that could be concealed:

“I have a colostomy bag, but it is not comparable. I can hide the bag; I can go to the beach or the swimming pool. But I can’t hide the psoriasis, and I’m fed up with what it has done to my hands.”

(P64, female, 42).

Furthermore, psoriasis affecting the genital region was described as particularly distressing because of the associated pain, itching, and impact on quality of life:

“When it appears in your private parts, well, it gets really bad. It itches, and it hurts.”

(P1, male, 74).

#### 3.3.2. Logistical Burden: The Demands and Frequency of Clinical Attendance

The requirement to attend phototherapy sessions three times per week was identified as a major source of stress and disruption to everyday life:

“Coming three times a week is too much; it’s a real struggle. I give up study time to come here, but I don’t see any results.”

(P6, female, 25).

This logistical burden frequently generated conflicts with employment responsibilities, leading to feelings of guilt toward colleagues and concerns about employers’ reactions:

“It’s frustrating; you feel an added burden, especially when you have to juggle it with work. Not all employers are understanding or willing to give you time off. Even though we have legal rights, you still feel pressured.”

(P52, female, 54).

For some participants, these structural barriers contributed to difficulties adhering to treatment schedules:

“This is a disaster [pointing to the phototherapy machines]. Between work and things at home, I haven’t been able to attend all the sessions I was supposed to attend.”

(P76, female, 56).

#### 3.3.3. Functional Impairment and the Pervasive Distress of Symptoms

Physical symptoms, particularly fissures and chronic itching, resulted in a profound sense of limitation and an inability to fulfil important occupational and family responsibilities. Participants with palmoplantar involvement frequently described feeling disabled by their symptoms:

“I feel disabled when that happens, and I have to work my entire shift with these wounds. I’m grateful that for the last eight years I haven’t needed to use my hands so much at work. But now I’ve lost my job and need to find a new one. I don’t know what I’ll find when every few days my hands are covered in cuts.”

(P63, male, 49).

The impact on family life was equally profound, affecting participants’ ability to engage in basic caregiving activities and maintain parental roles:

“It has affected me a lot. For example, I wasn’t able to hold my daughter’s hand while walking her to school. She was five years old when my psoriasis was at its worst. I couldn’t help her with her hygiene or comb her hair. I have to delegate tasks that are usually simple for me, but sometimes I just can’t do them.”

(P56, female, 43).

This functional deterioration often contributed to social withdrawal and isolation:

“I barely go out, except to walk my dog. Some mornings I can’t even get out of bed. My feet are covered in cuts; they bleed, they itch, it’s horrible. I really don’t know how to cope with what is happening to me.”

(P28, female, 50).

This cycle of distress was further intensified by chronic itching, which influenced sleep, daily functioning, and medication use:

“Taking pills at night to help me sleep, and then another pill with the opposite effect to help me wake up.”

(P11, female, 62).

Participants also described the embarrassment associated with scratching in public:

“I’m afraid people will think I have lice.”

(P43, male, 60).

### 3.4. Thematic Area 4: Healthcare Expectations: Barriers and Proposals for Service Improvement

The cumulative impact of these functional stressors and biographical disruptions ultimately shaped participants’ expectations regarding healthcare delivery and service improvement. This final thematic area explores participants’ perceptions of existing structural barriers and highlights the need for more integrated, patient-centred, and nurse-led models of care.

#### 3.4.1. Structural and Social Barriers: Diagnostic Delays and Stigma

Participants identified significant gaps within the healthcare pathway, beginning with delays in diagnosis that they attributed to a perceived lack of psoriasis-specific knowledge in primary care settings:

“When you go to the health centre, general practitioners do not know much about psoriasis. I think it is something unfamiliar to them. I was referred there [to the dermatologist] because I started crying during the consultation.”

(P61, male, 30).

Participants also perceived that psoriasis continued to be viewed as a predominantly cosmetic condition, resulting in inequalities in access to and funding of essential healthcare resources:

“The healthcare system still considers psoriasis to be a cosmetic disease. That is my perception because I have had to pay for everything I have needed, apart from creams containing corticosteroids, while other treatments that are truly aesthetic are covered…”

(P11, female, 62).

#### 3.4.2. The Healthcare Professional as a Key Reference Point for Integrated Care

Participants emphasized the need for a dedicated healthcare professional who could provide guidance and continuity throughout the treatment process. Within their experiences of phototherapy, the nurse was frequently described as a trusted and accessible point of reference.

Participants also highlighted the importance of having dedicated nursing consultation rooms that would provide the privacy required to address both clinical and psychosocial concerns:

“It would be ideal to have a nursing consultation room exclusively for psoriasis patients; that would be wonderful. Here, we end up sharing consultation rooms with other patients, and we do not have the level of privacy that we need.”

(P11, female, 62).

Similarly, participants valued phototherapy not only because of its dermatological benefits but also because it provided regular access to professional guidance and emotional support from the nurse:

“Coming to phototherapy and having time to ask the nurse questions has helped me improve the psychological aspect of my disease. She helps you; she is someone you can rely on, and that is something I value very highly.”

(P56, female, 43).

## 4. Discussion

This study revealed the multifaceted burden and logistical tensions inherent in psoriasis management, highlighting the conflict between clinical efficacy and the daily disruption caused by phototherapy. While the general psychosocial impact of psoriasis is well-documented, these findings illuminate the specific and under-researched logistical challenges of the phototherapy regimen, revealing how its demanding frequency can paradoxically exacerbate the patient’s stress and perceived incapacity.

### 4.1. Health Literacy and Therapeutic Perceptions: Addressing the Systemic Knowledge Gap

Participants demonstrated limited knowledge regarding several fundamental aspects of psoriasis and its treatment, although a clear dichotomy emerged. While they displayed a strong awareness of the psychological consequences of the disease, often perceiving anxiety and depression as integral components of their experience, they remained largely unaware of its associated physical comorbidities.

This finding is consistent with the study by Cingöz et al., who reported that although patients demonstrated adequate awareness of the psychological consequences of psoriasis, knowledge regarding other important aspects of the disease, such as increased cardiovascular risk, remained limited [28]. Similarly, Zhang et al., in a recent study exploring knowledge, attitudes, and practices among people with psoriasis, found that despite significant clinical advances, patients continue to demonstrate insufficient knowledge regarding disease triggers, genetic factors, and the potential adverse effects of systemic treatments [18].

Although these findings are broadly consistent with the existing literature, previous studies have primarily examined this issue from a quantitative perspective, providing only a limited understanding of patients’ subjective experiences. In contrast, qualitative inquiry offers a deeper exploration of how these knowledge gaps are experienced and interpreted by individuals living with psoriasis.

Consistent with the findings of the present study, González et al. identified important deficiencies in participants’ understanding of disease pathophysiology and clinical manifestations, with many individuals maintaining misconceptions regarding disease contagion [17]. Interestingly, despite these knowledge gaps, participants in the study by González et al. expressed generally favourable attitudes towards biological therapies. This contrasts with the findings of the present study, where perceptions of biological treatments were predominantly negative and were strongly influenced by concerns regarding the potential risk of developing cancer.

The clinical relevance of addressing these knowledge deficits is further supported by Tian et al., who demonstrated a significant positive association between higher levels of disease-related knowledge and more favourable attitudes towards treatment, as well as more proactive self-management behaviours [19]. These findings suggest that reducing existing information gaps may represent an important step towards promoting patient empowerment and strengthening self-efficacy.

In this regard, evidence from a recent systematic review indicates that targeted nursing interventions, particularly those focused on structured patient education and self-management support, play a fundamental role in improving health-related quality of life and treatment adherence among people with psoriasis [16].

### 4.2. The Adherence-Stress Paradox: Phototherapy as a Double-Edged Sword

The findings of this study highlight a critical paradox in the management of psoriasis through phototherapy. While phototherapy is an effective treatment for improving clinical outcomes, it may simultaneously function as an additional source of stress, thereby contributing to increased psychological burden. This “adherence–stress paradox” emerged from participants’ accounts of how the rigid requirement to attend treatment sessions three times per week generated the very stress that many identified as a primary trigger for disease exacerbation.

These findings are consistent with the international literature, which characterizes phototherapy as an effective but inherently demanding therapeutic modality [29,30]. The success of phototherapy is highly dependent on adherence to a rigorous treatment schedule, as emphasized in international clinical guidelines [14]. These guidelines acknowledge that logistical barriers and the frequency of required clinical visits are among the most important factors influencing treatment adherence.

The present findings extend this existing knowledge by demonstrating how these logistical demands are experienced by patients in their everyday lives. Participants described the treatment schedule as a significant source of disruption, affecting their ability to fulfil professional, family, educational, and social responsibilities. Consequently, the burden associated with treatment extends beyond the clinical setting and becomes embedded within patients’ broader life narratives.

The present study provides an in-depth illustration of treatment burden, identifying it as a key factor that may negatively affect overall quality of life. Although the logistical demands of attending phototherapy sessions three times per week are specific to this treatment modality, they may also serve to magnify broader challenges associated with living with a chronic disease such as psoriasis. Unlike more discreet systemic therapies, phototherapy requires repeated attendance at healthcare facilities, compelling patients to confront the visibility and chronicity of their condition on a regular basis.

Therefore, treatment burden should not be understood solely as a technical or organizational challenge. Rather, it represents an intensified manifestation of the broader biographical disruption associated with psoriasis. The findings suggest that the impact of treatment extends beyond symptom management and becomes deeply intertwined with patients’ identities, daily routines, and social roles.

### 4.3. Biographical Disruption and the Social Stigma of Visibility

The narratives collected in this study provide a compelling illustration of the concept of biographical disruption, whereby psoriasis extends beyond a physical condition and profoundly affects an individual’s identity, social relationships, and everyday functioning [10]. Participants frequently described how the visibility of their lesions, particularly in exposed body areas, forced them to renegotiate their sense of self and their interactions with others.

The feelings of embarrassment, shame, and fear of negative judgement reported by participants closely mirror the findings of Moschogianis et al., who described how social interactions often become unwelcome reminders of the disease and contribute to feelings of vulnerability and altered self-perception [31]. These authors further introduced the concept of the “threatened self,” whereby chronic skin disease challenges an individual’s social identity and self-worth [31]. Consistent with this perspective, the present findings suggest that psoriasis-related stigma extends beyond social discomfort and may profoundly affect how individuals perceive themselves within their family, social, and occupational roles.

Importantly, the findings demonstrate that this disruption is not confined to public settings but also affects the most intimate aspects of daily life. Participants described being unable to perform simple acts of caregiving and affection, such as holding a child’s hand or assisting with personal hygiene. These limitations represent more than functional restrictions; they signify a disruption of valued social identities, particularly those associated with parenting and caregiving responsibilities.

In this context, the skin becomes more than a visible manifestation of disease. It may function as a physical and symbolic barrier that interferes with the fulfilment of everyday social roles and meaningful interpersonal relationships. Consequently, the burden of psoriasis extends beyond symptom severity and becomes deeply embedded within patients’ lived experiences.

Similarly, the feelings of disability and reduced occupational capacity reported by participants with palmoplantar involvement are consistent with the findings of González et al., who identified psoriasis as a significant barrier to employment stability and professional continuity [17]. The fear of being unable to obtain or maintain employment because of recurrent skin lesions illustrates how psoriasis may disrupt not only present-day functioning but also future life plans and expectations. These findings reinforce the notion that psoriasis should be understood not solely as a dermatological condition but as a chronic illness with substantial psychosocial, relational, and occupational consequences.

### 4.4. Implications for Nursing: The Demand for a Specialized Care Model

The findings of this study reveal a perceived lack of continuity and support throughout the healthcare journey of people living with psoriasis. Participants consistently expressed the need for a dedicated healthcare professional who could provide guidance, continuity, and support across different stages of disease management. Their accounts suggest that current models of care do not always adequately address the psychosocial and practical challenges associated with living with psoriasis.

A particularly important finding was the value participants attributed to the nurse as a trusted and accessible point of contact. The demand for a dedicated nursing professional, as well as for private consultation spaces, reflects a broader need for more personalized and patient-centred models of care. Participants described nurses not only as providers of treatment but also as sources of emotional support, health education, and practical guidance.

Although contemporary healthcare increasingly embraces multidisciplinary approaches, participants’ experiences suggest that dermatology nurses may play a uniquely important role in coordinating care and supporting patients throughout the health–illness process. This role extends beyond the technical administration of treatment and encompasses the management of psychosocial concerns, treatment-related challenges, and health literacy needs.

The sense of abandonment reported by some participants suggests that traditional models of care may continue to prioritize clinical outcomes over the psychosocial and logistical difficulties associated with psoriasis. These findings support the growing recognition that effective psoriasis management requires a holistic approach that integrates both physical and psychological dimensions of care.

Consistent with this perspective, Al-Hayti et al. argued that the inclusion of dermatology nurses within multidisciplinary teams is not merely complementary but represents a fundamental component of high-quality psoriasis care, contributing significantly to improved quality of life and treatment outcomes [16].

Within phototherapy units, the role of the nurse should therefore evolve beyond the technical administration of treatment toward the provision of comprehensive patient support. The findings indicate that patients require ongoing guidance to manage treatment burden and cope with the adherence–stress paradox identified in this study. In particular, specialized nursing support may be essential for addressing misconceptions about psoriasis, improving health literacy, and reducing barriers to treatment adherence.

Furthermore, participants’ narratives suggest that nurse-led models incorporating structured education, emotional support, and individualized follow-up may help address unmet needs related to functional impairment, social isolation, and psychological distress. Such interventions have the potential not only to improve treatment adherence but also to reduce the overall burden of living with psoriasis and promote a more integrated and patient-centred model of care.

### 4.5. Implications for Policy and Practice

A major strength of this study is its large sample size for a qualitative investigation, which enabled the identification of recurring patterns across a broad range of heterogeneous life circumstances. The inclusion of participants with diverse demographic, occupational, and clinical backgrounds enriched the dataset and facilitated a robust cross-case analysis. This diversity strengthened the interpretation of the findings and provided a comprehensive understanding of the treatment burden associated with psoriasis and phototherapy.

Furthermore, the large sample allowed the research team to explore the phenomenon across multiple life contexts, enhancing the richness and breadth of the data. The consistency of themes across participants with different experiences and backgrounds increased confidence in the robustness of the findings and supported the development of broader conceptual interpretations.

Nevertheless, it is important to acknowledge that the breadth afforded by a large sample may, in some instances, limit the level of depth that can be achieved for individual narratives. While the study sought to maintain analytical depth through iterative and detailed analysis, smaller phenomenological studies may provide a more nuanced exploration of highly individual and idiosyncratic experiences.

The findings should also be interpreted within their specific cultural and healthcare context. As the study was conducted exclusively in Spain, participants’ experiences of healthcare delivery, social stigma, and disease management are inevitably influenced by the characteristics of the Spanish healthcare system and the surrounding sociocultural environment. Consequently, the transferability of these findings to other healthcare settings and cultural contexts should be considered with caution.

For example, perceptions of access to healthcare services, continuity of care, and relationships with healthcare professionals may differ substantially in countries with alternative healthcare structures. Likewise, cultural attitudes towards visible skin conditions may influence experiences of stigma and social exclusion.

An additional limitation relates to the extended period of data collection, which took place between 2019 and 2022 and therefore overlapped with the COVID-19 pandemic. This context may have influenced participants’ experiences of healthcare access, social isolation, and psychological distress. However, the consistency of the thematic findings throughout the study period suggests that the core experiences associated with treatment burden and disease management remained stable despite these unprecedented societal challenges.

Future research should incorporate longitudinal designs to examine how treatment burden, coping strategies, and disease management experiences evolve over time. In addition, multicentre studies conducted across different healthcare systems and cultural settings would help identify both universal and context-specific barriers to effective psoriasis management.

Further research is also needed to evaluate the effectiveness of specific nursing interventions aimed at improving health literacy, addressing misconceptions about psoriasis, reducing treatment burden, and facilitating the integration of disease management into patients’ personal and professional lives. Such work may contribute to the development of more effective, integrated, and patient-centred models of care for people living with psoriasis.

## 5. Conclusions

This study identifies a critical tension between clinical efficacy and treatment burden in the management of psoriasis through phototherapy. Although this therapeutic modality is safe and effective, its demanding treatment schedule often functions as a double-edged sword, whereby the logistical challenges of balancing treatment with work, family, and social responsibilities generate chronic stress that may paradoxically exacerbate disease activity.

Furthermore, the findings reveal a persistent knowledge gap regarding the systemic comorbidities associated with psoriasis, leaving patients vulnerable to misconceptions and limiting their capacity for effective self-management. Beyond the physical manifestations of the disease, the anatomical location of lesions contributes to profound biographical disruption, affecting individuals’ identities and their ability to fulfil professional, social, and intimate roles.

Ultimately, these findings suggest that achieving clinical skin clearance alone may be insufficient if the broader psychosocial, educational, and systemic challenges associated with psoriasis remain unaddressed. This study supports a shift towards a more integrated model of care within dermatology services, one that recognizes the complex interaction between disease burden, treatment demands, and patients’ lived experiences.

In this context, the implementation of specialized dermatology nursing roles may be essential to provide continuity of care, facilitate clinical navigation, improve health literacy, and deliver the emotional support required to manage the health–illness process beyond the surface of the skin.

## Figures and Tables

**Table 1:** Characteristics of the participants.

Characteristics	*n* (%) or Mean ± SD
**Age (years)**	46.5 ± 15.1 (19–76)
**Gender:**	
- Male	31 (43%)
- Female	41 (57%)
**Marital Status:**	
- Married/stable relationship	52 (71%)
- Divorced, widowed or single,	21 (29%)
**Employment status:**	
- Active	42 (58%)
- Without job	9 (13%)
- Student	3 (4%)
- Retired	18 (25%)
**Mental illness**	
- Yes	17 (24%)
- No	55 (76%)
**Difficulty of going therapy**	
- No	57 (79%)
- Yes	15 (21%)

## Data Availability

The data presented in this study are available upon request from the corresponding author due to privacy/legal/ethical reasons.
